# Supplementary figures and images for: Ursolic Acid Alleviates Neuroinflammation after Intracerebral Hemorrhage by Mediating Microglial Pyroptosis via the NF-κB/NLRP3/GSDMD Pathway
Source: Int J Mol Sci. 2023 Sep 30;24(19):14771. doi: 10.3390/ijms241914771 (PMC10572659; doi:10.3390/ijms241914771)

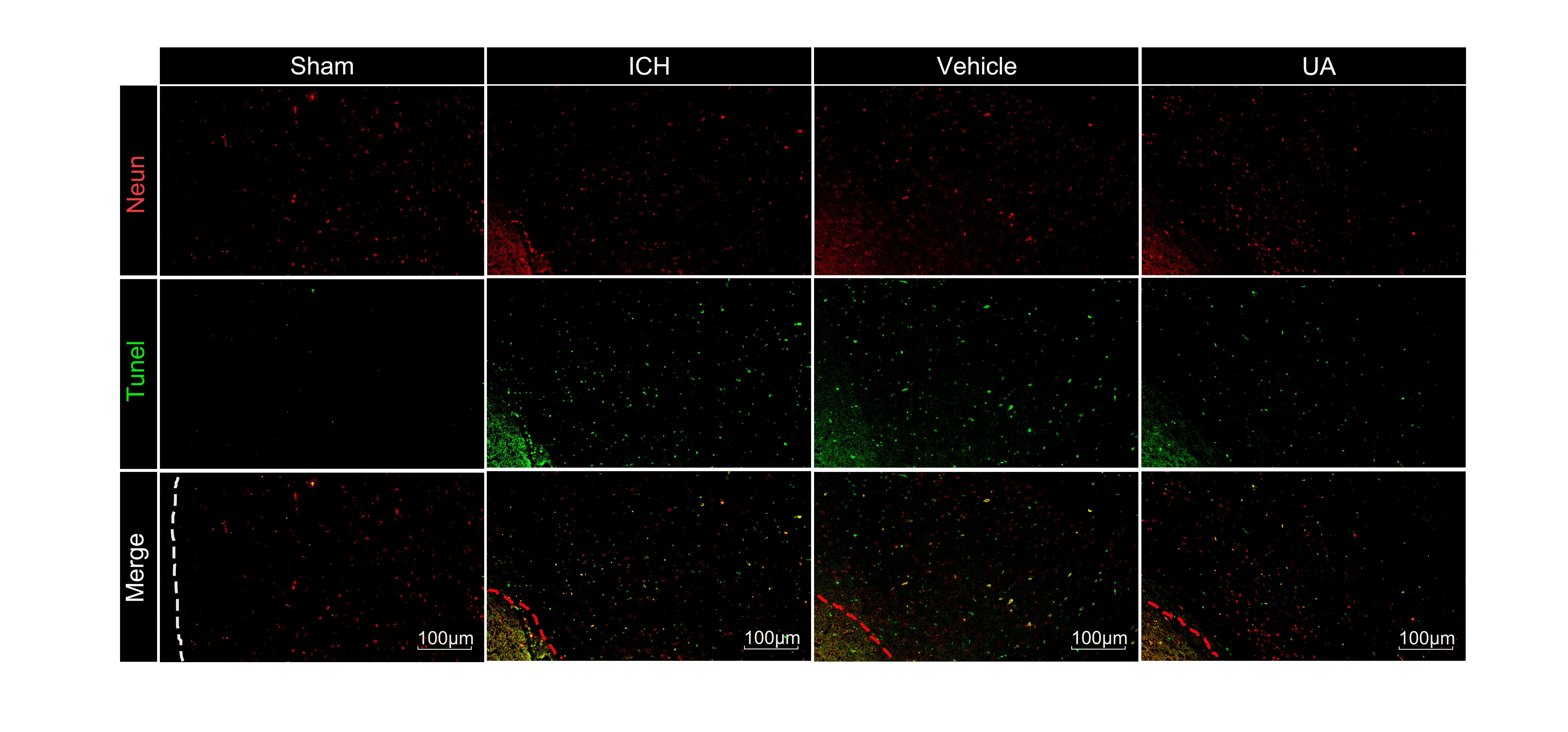

Supplement: Supplementary file 1 [file ijms-24-14771-s001.zip › Supplemental Figure S1.tif]

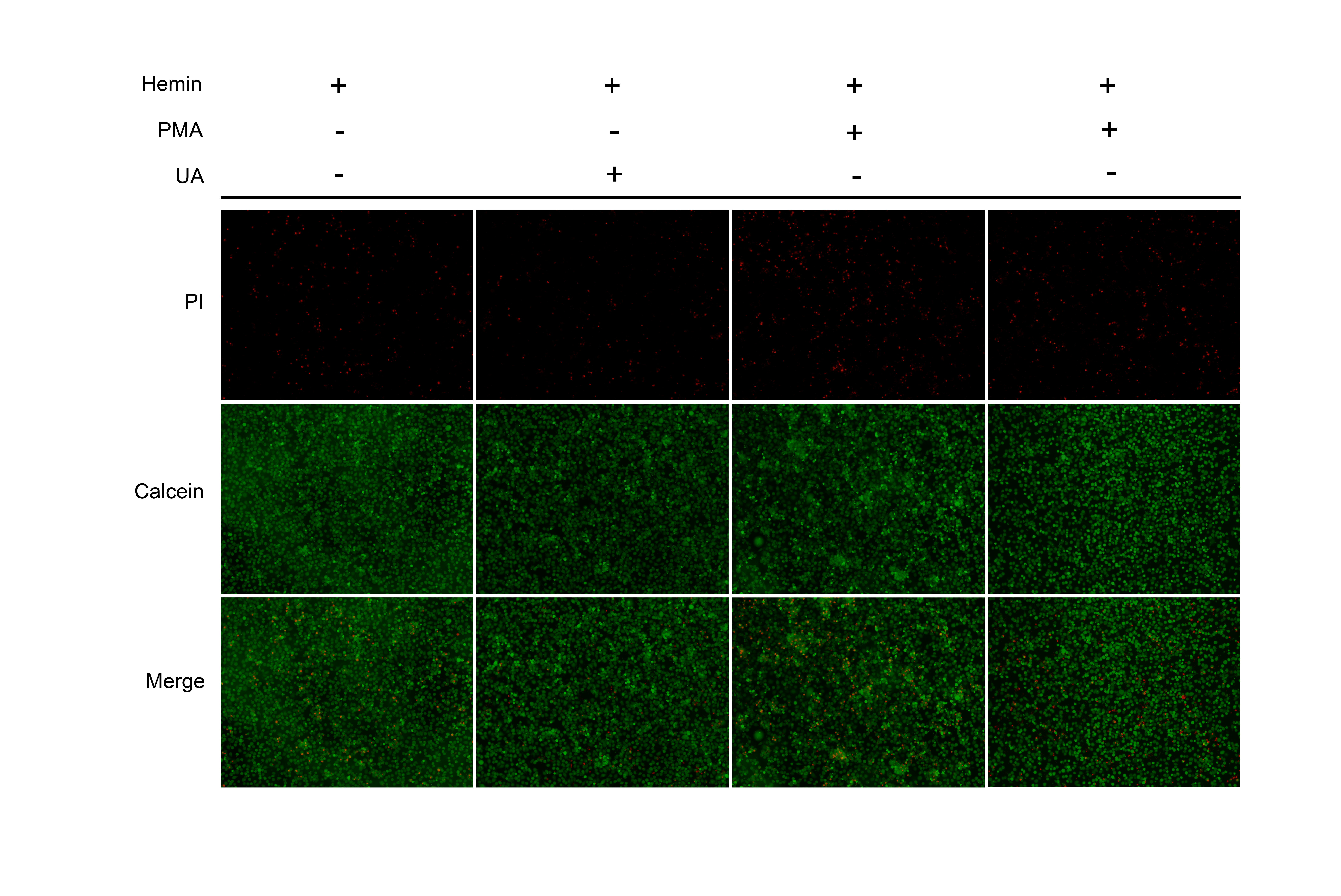

Supplement: Supplementary file 1 [file ijms-24-14771-s001.zip › Supplemental Figure S2.tif]
